# Siamese Architecture-Based 3D DenseNet with Person-Specific Normalization Using Neutral Expression for Spontaneous and Posed Smile Classification

**DOI:** 10.3390/s20247184

**Published:** 2020-12-15

**Authors:** Kunyoung Lee, Eui Chul Lee

**Affiliations:** 1 Department of Computer Science, Graduate School, Sangmyung University, Hongjimun 2-Gil 20, Jongno-Gu, Seoul 03016, Korea; guy9284@gmail.com; 2 Department of Human-Centered Artificial Intelligence, Sangmyung University, Hongjimun 2-Gil 20, Jongno-Gu, Seoul 03016, Korea

**Keywords:** smile classification, affective computing, automatic facial expression analysis, 3D CNN, deep metric learning

## Abstract

Clinical studies have demonstrated that spontaneous and posed smiles have spatiotemporal differences in facial muscle movements, such as laterally asymmetric movements, which use different facial muscles. In this study, a model was developed in which video classification of the two types of smile was performed using a 3D convolutional neural network (CNN) applying a Siamese network, and using a neutral expression as reference input. The proposed model makes the following contributions. First, the developed model solves the problem caused by the differences in appearance between individuals, because it learns the spatiotemporal differences between the neutral expression of an individual and spontaneous and posed smiles. Second, using a neutral expression as an anchor improves the model accuracy, when compared to that of the conventional method using genuine and imposter pairs. Third, by using a neutral expression as an anchor image, it is possible to develop a fully automated classification system for spontaneous and posed smiles. In addition, visualizations were designed for the Siamese architecture-based 3D CNN to analyze the accuracy improvement, and to compare the proposed and conventional methods through feature analysis, using principal component analysis (PCA).

## 1. Introduction

As indicators of human emotions, facial expressions are the most informative nonverbal behavior. Furthermore, as autonomic nervous system responses, facial movements are autonomously expressed, even when unintended. In other words, facial expressions are powerful visual indicators of the emotions and psychological state of individuals [1]. Consequently, facial expressions and movements have been studied in various fields, including biometric authentication, forensic science, and diagnosis of disorders [2,3,4]. In this study, we investigated spontaneous and posed smile classification, which is one way of inferring human psychological states through facial expression analysis. A spontaneous smile is a smile produced by emotion. In other words, it is a smile that is expressed autonomously by emotional stimulation, such as watching a comedy video. In contrast, a posed smile does not correspond to emotion, instead it is induced by a verbal command. Moreover, a neutral expression is a face in a non-expressive state, in which the emotional state is not revealed [5]. In addition, the use of the neutral expression as a standard for facial expression measurement is a well-known method in face analysis, but there has been no attempt to classify the two smiles using a Siamese architecture of deep learning approaches [6].

Differences between these two types of smile have been found in clinical neuroscience studies [7]. For instance, a Parkinson’s disease patient group exhibited fewer spontaneous facial expressions than the control group. In addition, some patients with Parkinson’s disease were unable to express spontaneous smiles, although they were able to express posed smiles. On the other hand, some patients with brain lesions could not form posed smiles, but could produce spontaneous smiles [8,9,10]. Patients with lesions in the motor cortex had difficulty expressing posed smiles. The posed smiles of these patients were asymmetrical, or they exhibited no movement of the lateral face on the side opposite the brain lesion, because the motor pathways cross over from one side of the brain to the other side of the body [11,12]. Patients with subcortical lesions showed the opposite symptoms. The posed smiles of these patients were normal, but their spontaneous smiles were abnormal. Obviously, these characteristics indicate that the two types of smiles are expressed through different motor pathways in the brain [13,14]. This motor pathway difference has elucidated the presence of six distinct facial motor cortex regions through anatomical observations of non-human primates, and to some extent, clinical observations. These different motor pathways, between posed and spontaneous expressions, cause spatiotemporal differences in facial movements. Ross and Pulusu et al. compared the movement–onset asymmetries of posed and spontaneous expressions, and confirmed that these differences explained up to 70% of the data variance, where the movement onset is a time series interval from the start of a facial expression until it reaches its apex. In addition, posed expressions started mainly from the right side of the face, whereas spontaneous expressions started mainly from the left side [12]. These differences enable the classification of posed and spontaneous smiles using machine learning.

Several machine learning models have been proposed that classify posed and spontaneous smiles using spatiotemporal differences in facial movements due to different hemispheric modulation. Most researchers have used image sequences that can extract temporal and spatial changes in facial movements, rather than only using the spatial information of facial expression images. Furthermore, most previous studies have involved the use of handcrafted feature extraction approaches to train machine learning models. However, with the recent advances and performance improvements of deep learning models, research on deep learning models for classifying the two smiles is indispensable [15,16]. In addition, by studying a deep learning model that includes, not only three-channel color images, but also hyperspectral imagery as inputs, it is possible to extract abundant features for classifying facial expressions [17].

In this study, we investigated the use of a 3D convolutional neural network (CNN) model to classify posed and spontaneous smiles. This approach was found to be suitable for solving the two-smile classification task because it could learn a descriptor that extracted spatiotemporal features, without domain knowledge about the differences between the two types of smile [16]. However, in our experiment, a 3D CNN alone did not show meaningful performance in classifying the subtle spatiotemporal differences of the two types of smiles. Therefore, we utilized a 3D CNN to apply a supervised metric-based approach, characteristic of a Siamese network. A Siamese network model learns to identify input pairs according to the probability that they belong to the same class or different classes [18]. Siamese networks are known to be capable of learning useful features for classification even when there are very few image samples in the classes. Since Siamese networks have superior generalized performance compared to CNNs alone, these networks have high accuracy, even for test data or new data. Hence, the abilities of Siamese networks were considered suitable for spontaneous and posed smile classification. We also employed new input pairs, including a neutral expression, to classify the two types of smiles, rather than using the posed and spontaneous smile input pair of a conventional Siamese network. Each input pair consisted of a neutral expression and a spontaneous smile, or a neutral expression and posed smile. This input pair configuration could mitigate individual differences due to variations in the shapes of the eyes, nose, and mouth between individuals, by learning through the input of neutral expression and smile video pairs. Variations in the shapes of facial features can cause differences in facial movements between individuals during facial expression, which is an important cause of model accuracy degradation. Hence, we addressed this problem by utilizing input pairs containing neutral expressions. In addition, the use of a neutral expression as an anchor image facilitated the development of a fully-automated spontaneous and posed smile classification system. If an input pair consisting of a genuine image and an imposter image is used, which is the conventional type of input pair for a Siamese network, the posed smile must be selected as the anchor image, which requires the intervention of trained experts to identify posed smiles. However, if a classifier is used with a neutral expression as the anchor image, this image can be selected as a median expression frame from data describing the degree of expression activation. This method was used because human expressions are mostly neutral in everyday situations, and median expression images have already been used in some algorithms [19]. In addition, Section 4.3 describes deep learning model feature analysis, using gradient-weighted class activation mapping (Grad-CAM) and principal component analysis (PCA) to assess the performance improvement of the proposed method. This paper is organized as follows: Section 2 covers related works on classifying two smiles. Section 3 describes data collection for training and evaluation of the deep learning model, and data preprocessing, such as face region segmentation, and includes implementation details for our proposed method. Section 4 covers the performance evaluation of our proposed method, performance comparison with other models, and visualization for analysis of the results. In Section 5, the experimental results are compared with related studies, and the limitations of this study and future works are discussed.

## 2. Related Works

### 2.1. Hand-Crafted Feature-Based Approaches

Over the past decade, many hand-crafted descriptors have been proposed for spontaneous and posed smile classification. Dibeklioğlu et al. [19] focused on eyelid movements to design a hand-crafted descriptor based on the observation made by Duchenne et al. [20], that the circular muscles around the eyes cannot be controlled in a posed smile. The authors employed distance- and angle-based features of the eyelids as descriptors in spontaneous and posed smile classification, using machine learning models such as the support vector machine (SVM), hidden Markov, k-nearest neighbor, and naive Bayesian classifier models. Their models classified the two types of smile with up to 91% accuracy. However, the results were obtained using a database that is not suitable for spontaneous and posed smile classification, and the number of samples was insufficient [19].

**Figure 1:**
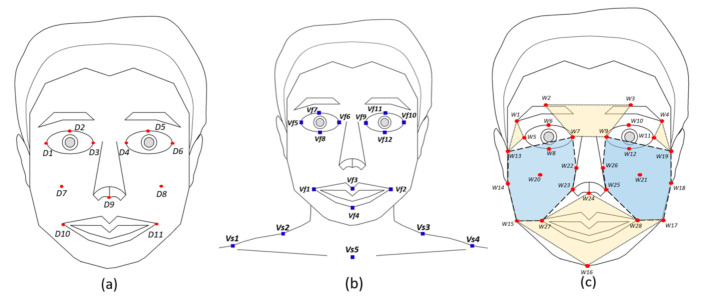
Feature points and facial regions used in hand-crafted feature-based approaches. (**a**) Dibeklioğlu et al. [21] used geometric features, based on 11 facial feature points (red points). (**b**) Valstar et al. [22] used geometric features, based on 12 facial feature points and 5 shoulder feature points (blue boxes). (**c**) Wu et al. [23] used geometric and appearance features, based on 28 facial feature points (red points) and the texture information in 6 facial regions (colored polygons).

Dibeklioğlu et al. [21] subsequently expanded their research to spontaneous and posed smile classification using features of the cheeks and mouth as well as eyelids. Figure 1 shows the facial feature points that they used to classify the two types of smile. These facial feature points were normalized according to 3D facial direction, and the features were extracted according to equations for calculating the amplitudes of the lip, eyelid, and cheek movements. In addition, the speed, duration, amplitude difference, and acceleration features were calculated based on the amplitudes of the facial movements. In this study, Dibeklioğlu et al. [21] achieved 87.82% classification accuracy using mid-level fusion SVMs on a database suitable for classification of the two types of smile, and which included 400 subjects.

Valstar et al. [22] extracted features related to not only facial muscle movements, but also head and shoulder movements. Figure 1 shows the facial and shoulder feature points used to classify the two types of smile in the related work. The authors measured the accuracy of their model using the MMI facial expression database [24]. This database consists of 2900 videos, showing the facial expressions of 75 subjects. Their model exhibited a classification accuracy of up to 94%. In their study, head movement features appeared to be as important in classifying the two types of smiles as facial expression movements [22].

Wu et al. [23] used the geometric features obtained from facial landmarks, as well as region-specific texture information, as hand-crafted features. The use of region-specific texture information involves extracting appearance features separately from the parts of the face corresponding to the forehead, cheeks, eyes, and mouth. Wu et al. [23] used a pixel-based approach, a Gabor filter, and a local binary pattern and histogram of oriented gradient (HOG) descriptors, which are traditional methods, to extract region-specific texture features. This method of SVM classifier training, using geometric features as well as appearance features, showed a state-of-art performance of 93.95%. This result demonstrates that geometric information, as well as appearance information, is important for machine learning models that classify spontaneous and posed smiles [23].

### 2.2. Deep Learning-Based Approaches

Mandal et al. [25] used CNN-based features for spontaneous and posed smile classification. They utilized not only CNN-based features, but also hand-crafted features to classify the two types of smile. The CNN used in this model was a visual geometry group network based face recognition model, which was pretrained for facial feature extraction by Parkhi et al. [26]. Mandal et al. [25] only used the CNN-based method for spatial feature extraction, and employed a hand-crafted method for temporal and spatial feature extraction. As hand-crafted feature extraction methods, a local phase quantization method for texture description [27], a HOG method for describing differentials between pixels [28], and a dense optical flow method for measuring the movement of fiducial points in an image were utilized [29]. This model classified the two types of smile using both hand-crafted and deep learning methods in the extraction of features as SVM inputs. This model achieved up to 78.14% accuracy. In addition, the experimental results showed that the feature information extracted using the HOG method was best in the most normalized environments. Although the HOG-based features appeared to achieve better classification performance than the CNN based-features, it is considered that this result occurred because the visual geometry group network was not suitable for spontaneous and posed smile classification.

Gan et al. [30] classified the two types of smile using a deep Boltzmann machine (DBM) model. They employed differential images as input to train the DBM. These images were difference images between the image of the smile apex and the image of the smile onset. The smile onset is the period from the moment at which a smile starts to the moment at which it reaches the peak. The smile apex is the period during which the smile is maintained after reaching the peak. The smile offset is the period from when the smile apex ends until the subject returns to a neutral expression. The authors trained a DBN that extracted spatiotemporal features through differential images between smile apex images and smile onset images. They measured the performance of the DBN on the natural visible and infrared facial expression database (USTC-NVIE) [31] and SPOS datasets [32], and the model accuracy was as high as 91.73% (USTC-NVIE) and 84.62% (SPOS), respectively.

Kumar et al. [33] designed and trained a CNN-based facial expression classification model. They reported that there was a difference between fake and spontaneous smiles using a prediction score extracted through the CNN model, and that this difference can be used for smile classification. However, the proposed model was intended to classify facial expressions corresponding to six basic emotions (happiness, sadness, disgust, anger, fear, and surprise) on the FERC-2013 dataset [34], and no attempt was made to prove or analyze the differences between fake and genuine smiles in the prediction score.

Yang, Y. et al. [35] proposed the first end-to-end deep learning model for the classification of two smiles. They proposed a fully-automatic method using a convolutional long short term memory network (ConvLSTM [36]), and confirmed the accuracy of the classification of two laughs, at 92%, on the MMI facial expression database [24]. In addition, this approach has the advantage of being able to input sequence frames of various lengths due to the recurrent neural network of ConvLSTM.

Wang, S. et al. [37] conducted the smile classification using a latent regression Bayesian network (LRBN). The proposed LRBN model detects patterns existing at facial landmark points, and learns the difference between spontaneous and posed smiles. This model attempted to classify posed and spontaneous facial expressions using only spatial patterns, and excluding temporal patterns, and found that facial spatial patterns were different according to gender and expression categories. This method showed 98.35% spontaneous and posed expression classification accuracy with the USTC-NVIE database [31].

## 3. Materials and Methods

### 3.1. Data Collection

We constructed a spontaneous and posed smile database to train the proposed 3D CNN model. The spontaneous and posed smile videos were obtained from 88 adults aged 20–80 years. While their facial movements were recorded with a camera, the participants watched a movie clip about 1 min-long to elicit happy emotions. The spontaneous smile videos were obtained by recording genuine smiles that appeared when the participants felt happy while watching a 1 min-long movie clip, while the posed smile videos recorded forced smiles that were induced through verbal commands without a feeling of happiness. Thus, the posed smiles were command-induced expressions, and the spontaneous smiles emerged spontaneously without any command. In addition, to acquire the neutral expressions used in our proposed method, we showed the participant a video without emotional stimulation, induced the participant’s non-expressive state, and acquired a neutral expression video. These smile videos were recorded by a Canon EOS 70D DSLR camera with a 50 mm prime lens, 720 p resolution, and 30 fps frame rate. All participants provided written informed consent prior to the experiment. This study was conducted in accordance with the declaration of Helsinki, and all experiments involving facial behaviors were approved by the institutional review board of the SMG-SNU Boramae medical center (irb no.30-2017-63).

### 3.2. Data Preprocessing

#### 3.2.1. Automatic Detection of Smile Moments

Each spontaneous smile video recorded in the experiments was 1-min-long in total. We extracted spontaneous smile moments from the recorded 1-min-long videos using the action unit (AU) detector provided in the OpenFace2.0 toolkit [38]. An AU is a unit of anatomy-based facial muscle movement taxonomy defined by the facial action coding system of Ekman et al. [11], and there are 28 main AUs that systematize major facial movements. The AUs for smile detection are AU06 and AU12, which correspond to cheek raiser (AU06) and lip corner puller (AU12) muscle movements. In this study, the AU detector of the OpenFace2.0 toolkit was used for automatic detection of smile moments. Both spontaneous and posed smiles were detected with the same smile detection threshold. The smile moments measured by automatic detection included 427 posed smile moments and 925 spontaneous smile moments. In addition, the spontaneous and posed smile videos were refined through visual inspection to confirm whether smile moments occurred.

#### 3.2.2. Facial Region Segmentation

**Figure 2:**
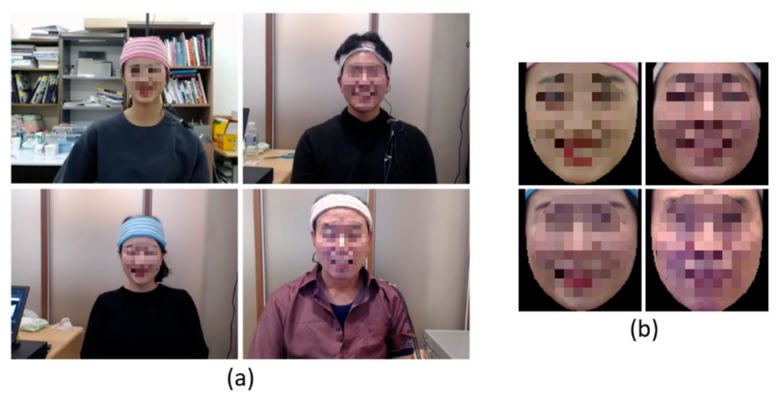
Examples of facial region segmentation. Smile videos (**a**) before facial region segmentation (1280 × 720-pixel resolution), and (**b**) after facial region segmentation (112 × 112-pixel resolution).

For efficient computation using the 3D DenseNet based on Siamese architecture, it was important to construct input videos that included facial regions but not background regions. Therefore, we used the OpenFace2.0 toolkit to remove the background regions from the original videos. Facial region segmentation was performed using the locations of facial landmarks corresponding to face outlines. Figure 2 shows the facial region segmentation results. By performing facial region segmentation, the original video with 1280 × 720-pixel resolution was cropped to a facial region video with 112 × 112-pixel resolution. This facial region segmentation could drastically reduce the computation and processing time of the 3D CNN model based on Siamese architecture.

#### 3.2.3. Time-Series Segmentation Using a Fixed-Size Sliding Window

To construct a dataset to train and evaluate the Siamese architecture-based 3D CNN model, time-series segmentation was conducted. Automatic detection of smile moments yielded 427 posed and 925 spontaneous smile moments. However, these numbers were too small to train a 3D CNN model and achieve generalized performance; hence, the time series dimension of the input images had to be fixed. The extracted smile moments were divided into spatiotemporal data with a fixed size sliding window of 1 s (30 frames), and the time series segmentation interval was 1 frame. For example, if time series segmentation was performed on a 2-s-long smile moment (60 frames), this moment was divided into 31 data with fixed spatiotemporal dimensions (112(width) × 112(height) × 30(time series domain)). The first datum corresponded to an image sequence in the 0–30 frame section, and the last datum composed an image sequence in the 30–60 frame section. Data augmentation using time-series segmentation with a fixed-size sliding window produced 24,492 spontaneous and 30,500 posed smile data. In addition, the dataset was separated based on the subject, to preserve the independence of the test, validation, and training data. Specifically, the data from 10 of the 88 total subjects were compiled into a test set, those from 10 other participants were used to form a validation set, and those from the remaining 68 participants were separated into a training set. Table 1 shows the details of the training, validation, and test sets, constructed through data preprocessing.

### 3.3. Method

#### 3.3.1. 3D DenseNet Based on Siamese Architecture

In this study, we developed a spontaneous and posed smile classification model using 3D DenseNet and Siamese architecture. Siamese networks are used for deep metric learning, and can measure the similarity between input pairs. Such networks have also achieved superior accuracy in signature and face verification, as well as many classification tasks [18]. A typical Siamese network measures the similarity between an anchor input and a paired input to determine whether the two inputs form a correct pair. However, our proposed model uses a neutral expression video as an anchor input, and a spontaneous or posed smile video as the paired input. This approach has several advantages in spontaneous and posed smile classification.

First, the fact that the proposed model does not use a posed smile as an anchor image greatly contributes to performance improvement. If the input pair of a common Siamese model is used for spontaneous and posed smile classification, either the spontaneous or posed smile must be used as the anchor image. However, in actual applications, using posed and spontaneous smiles as anchor images requires expert intervention to differentiate between the two types of smiles, which can hinder the automation of spontaneous and posed smile classification. Meanwhile, in the proposed model, there is no need for expert intervention, because a neutral expression is used as the anchor. A neutral expression can be extracted through median selection [38], which is advantageous for automation.

Second, this approach can normalize person-specific effects in the facial movements that occur while forming a smile due to individual differences in facial appearance. The neutral expression used as an anchor input serves as a reference for this normalization. This model classifies the two types of smiles according to the spatiotemporal differences between the neutral expression and the two types of smile; therefore, it is possible to extract spatiotemporal features focusing on facial movements rather than facial appearance.

Third, the powerful discriminative features of the Siamese network enable the proposed model to generalize a high predictive power for the smile of a person with a new facial appearance that is yet to be learned. Siamese networks learn the distance metric between classes, rather than learning the distribution of the classes; therefore, it is possible to generalize new data [14]. This approach makes it possible to train a model with only a few smile samples per subject. Furthermore, the proposed model can generalize the posed, spontaneous smile of a new person without having to undergo online learning. This not only contributes to the full automation of the spontaneous and posed smiles classification task, but also contributes a generalization with high predictive power to the two smile classification application.

**Figure 3:**
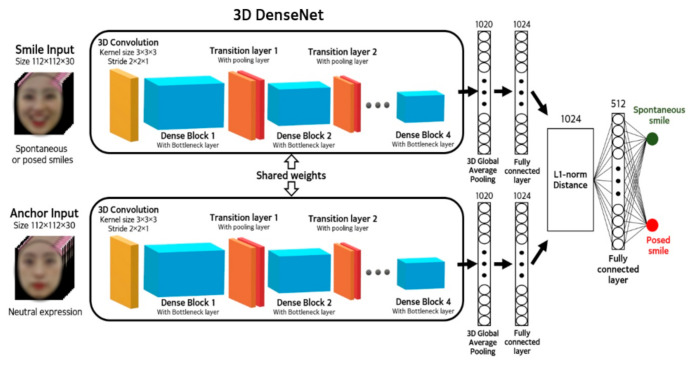
Overview of the Siamese architecture-based 3D DenseNet with person-specific normalization using neutral expression for spontaneous and posed smile classification.

Figure 3 shows an overview of the proposed model. Two 3D DenseNet pairs with identical weight parameters are employed to extract the spatiotemporal features of a spontaneous smile, posed smile, and neutral expression. Using these network pairs with the same weight parameter, the feature vectors of the anchor and smile inputs are extracted. Subsequently, the distance between these two feature vectors is measured, according to the L1-norm distance. In a fully-connected layer, these distances serve as metrics for classification of the smile input, as either spontaneous or posed.

#### 3.3.2. Model Structure

A 3D DenseNet can perform advanced feature extraction with fewer model parameters compared to other networks. Moreover, due to the structure of DenseNet, which feeds forward the concatenation of the input and output feature maps, it has a high feature extraction performance and network classification accuracy. Since the 3D convolutions of a 3D CNN are computationally expensive, a 3D Siamese DenseNet with fewer parameters is suitable for spontaneous and posed smile classification. In addition, all dense blocks in this 3D Siamese DenseNet model have a bottleneck layer to improve the computational efficiency. The bottleneck layer uses 3D convolution, with 1 × 1 × 1 filter size, effectively reducing the depth of the feature map, which is increased by concatenation. Furthermore, the bottleneck layers all go through batch normalization and a rectified linear unit activation layer before reaching the subsequent convolutional layer. In addition, the transition layers reduce the spatiotemporal dimensions of the feature maps through 3D average pooling.

Table 2 describes the details of the structure in the proposed 3D Siamese DenseNet architecture with 96 convolutional layers. This network structure consists of dense blocks, with bottleneck and transition layers. The transition layers differ depending on the type of pooling layer. There are two types of transition layers. In the first type, both the spatial and temporal dimensions are reduced by the pooling layer. In the second type, only the spatial dimension is reduced by the pooling layer. The reason that the pooling layers of the transition layers have different strides is the information loss problem of the temporal domain, which has fewer dimensions than the spatial domain. The spatial dimensions of the network input are 115 × 115, whereas the temporal dimension is only 30. When dimension reduction is performed with the same stride size in each pooling layer, the temporal dimension becomes so small that important information is lost. The performance of the 3D Siamese DenseNet is improved when the spatial and temporal dimensions are accurately adjusted, so that the network can learn spatiotemporal feature extraction.

As shown in Figure 3, the smile and anchor input videos are passed through two dense 3D CNNs, and features are extracted to form 1020 1D vectors through the 3D average pooling layer. Two averaged pooled 1D vectors are not directly used for distance calculations in deep metric learning. We placed a 1024-dimensional fully-connected layer between the metric calculation and average pooled 1D vector, and found the classification accuracy with the additional fully-connected layer was higher than that obtained by performing the metric calculation using the 3D average pooling result. Finally, the 3D Siamese network classifies the two types of smile by calculating the L1-norm distance between the two feature vectors computed through the two 3D DenseNet sharing the weights. The final fully-connected, layer determines whether the paired input is a posed smile, based on the calculated distance vector. After building the 3D Siamese DenseNet model, the total number of weight parameters was found to be approximately 6.4 million. Additionally, in the environment using two GTX 1080 TI graphics cards, the inference time per sample of the proposed model was about 46 ms.

#### 3.3.3. Loss Function and Parameter Initialization

The input of a typical Siamese network consists of a genuine pair, with the anchor and paired inputs in the same class, and an imposter pair with the anchor and paired inputs in different classes. In the conventional Siamese network, the loss function is designed so that the distance between the feature vectors of the inputs of a genuine pair is smaller, and the distance between the feature vectors of the inputs of an imposter pair is larger. These types of loss functions include triplet and contrastive loss functions. However, since the input pair containing a neutral expression in the proposed model is not a genuine or imposter pair, we used the following regularized cross-entropy loss function so that the model learns the distance between the posed smile and the neutral expression, and the spontaneous smile and the neutral expression:

(1) $$L\left(D\right)=\frac{1}{N}\;\underset{i\in D}{\sum }\left(\;y\left(a^{i},s^{i}\right)\log P\left(a^{i},s^{i}\right)+\;\left.\left(1-y\left(a^{i},s^{i}\right)\right)\log\left(1-P\left(a^{i},s^{i}\right)\right)+\;\alpha {\left\|w\right\|}^{2}\right)\right.,$$

where D is the entire index of the minibatch, N means the number of samples corresponding to D, i is the index of a sample corresponding to D, $y\left(a^{i},s^{i}\right)$ is the class of the anchor and smile input pair corresponding to the ith sample of D, and $P\left(a^{i},s^{i}\right)$ is the prediction result of the anchor and smile input pair, corresponding to the ith sample of D in the 3D Siamese DenseNet model (Figure 3).

The weight initialization method utilized for the 3D Siamese DenseNet is the same as that employed by Koch et al. [14]. The weights of the network are zero-mean normal distributions with standard deviations equal to 1/fan-in. Here, fan-in is defined as the number of weights received for each neuron in each layer of the network. The biases are initialized following Gaussian distributions, with a mean of 0.5 and fixed variance of $10^{-2}$. In addition, dropout regularization is performed for each bottleneck layer, and the dropout rate is set to 0.5.

## 4. Experimental Results

### 4.1. Performance of the Proposed Model

In this experiment, we measured the performance of the proposed model on a database, for which the data collection in Section 3.1, and preprocessing in odel on a database, for which the data collection in Section 3.2, were conducted. This database consists of the spontaneous and posed smiles of 88 subjects, including 24,492 spontaneous and 30,500 posed smile data. For correct performance measurement, the data of 68 subjects were used as training data, those of 10 subjects were used as validation data, and those of 10 subjects were used as test data. In addition, five-fold cross validation was performed for the efficient use of the data of 78 people excluding the test dataset. The number of subjects for validation was set to 10, and test accuracy was measured based on the most appropriate model parameter and hyper parameter in five-fold cross validation. The performance measurement indicated that the spontaneous and posed smile classification accuracy of the proposed model was 98.12%. Table 3 shows the details of the performance of the proposed model, with various networks and methods. In Table 3, we compare the 3D DenseNet models with various network depths, as well as existing studies trained and evaluated on the same database to evaluate the performance of the proposed model. Lee et al. [39] presented a score-level fusion method in which the output of a temporal stream convolutional network [40] receives optical flow outputs as inputs, and a support vector regression model receives facial spatial and facial asymmetry information. Section 4.2 presents visual results obtained using Grad-CAM [41], which were utilized to check the spatiotemporal features extracted from the proposed model. In addition, when using two GTX 1080 TIs, the model inference time was about 46 ms.

### 4.2. Visual Explanations Using Grad-CAM for Siamese Architecture-Based 3D CNN

Since a 3D CNN has activation values for both spatial and time series information, a Grad-CAM algorithm suitable for a 3D CNN was required. This section describes the feature extraction of the 3D CNN using Grad-CAM visualizations in the 2D spatial and time series domains. The target layer for extracting the activation map to perform Grad-CAM is the last convolutional layer of the 3D DenseNet. This layer belongs to dense block 4 in Figure 3. The output of this target layer ($A^{k}\;\in \;{\mathbb{R}}^{u\;\times \;v\;\times w}$) is a 3D tensor composed of width (u), height (v), and time series (w) axes. To calculate the 2D class activation map ($L_{spatial\;Grad-CAM}^{c}\in \;{\mathbb{R}}^{u\;\times \;v}$) in the spatial domain, we projected each 3D output tensor into a 2D domain (u, v) using the average operation on the time series axis. The equation for calculating $L_{spatial\;Grad-CAM}^{c}$ is as follows:

(2) $$\alpha_{k}^{c}=\frac{1}{Z}\underset{u}{\sum }\underset{v}{\sum }\;\frac{1}{w}\underset{w}{\sum }\frac{\delta y^{c}}{\delta A_{u,v,w}^{k}}\;\;,\;L_{spatial\;Grad-CAM}^{c}=ReLU\left(\underset{k}{\sum }a_{k}^{c}\;\frac{1}{w}\underset{w}{\sum }A_{u,v,w}^{k}\right)$$

The neuron importance weights, $\alpha_{k}^{c}\;\in \;{\mathbb{R}}^{u\;\times \;v}$, indicate the importance of feature map, k, for target class, c. As it is averaged based on the time series (w) axis and projected in the 2D domain (u, v), $L_{spatial\;Grad-CAM}^{c}$ is the activation map for class c in the 7×7 spatial domain of the target layer output space. The equation for calculating the 2D class activation map ($L_{time\;series\;Grad-CAM}^{c}\in \;{\mathbb{R}}^{v\;\times \;w}$) in the time series domain is similar to Equation (2). We projected each 3D output tensor into a 2D domain by averaging the width axis. The equation for calculating $L_{time\;series\;Grad-CAM}^{c}$ is as follows:

(3) $$\alpha_{k}^{c}=\frac{1}{Z}\underset{v}{\sum }\underset{w}{\sum }\;\frac{1}{u}\underset{u}{\sum }\frac{\delta y^{c}}{\delta A_{u,v,w}^{k}}\;\;,\;L_{time\;series\;Grad-CAM}^{c}=ReLU\left(\underset{k}{\sum }a_{k}^{c}\;\frac{1}{u}\underset{u}{\sum }A_{u,v,w}^{k}\right)$$

Only the axis of the average operation used to project the 3D tensor onto the 2D plane is changed, while the rest of the operation is the same as in Equation (2). The 2D axes of $L_{time\;series\;Grad-CAM}^{c}$ are the height and time series axes. However, there are two Grad-CAM results because there are two pairs of 3D CNN models in the Siamese network. A Siamese network calculates metric features through the L1-norm distance between the feature vectors extracted from two models with equal weights, therefore we designed the Grad-CAM for the Siamese architecture-based 3D CNN in this manner, as shown in Equation (4):

(4) $$L_{Grad-CAM\;for\;SNet}^{c}=\left|\;L1\;distance\left(L_{Grad-CAM\;on\;R\;model}^{c}\;,\;L_{Grad-CAM\;on\;L\;model}^{c}\right)\;\right|$$

**Figure 4:**
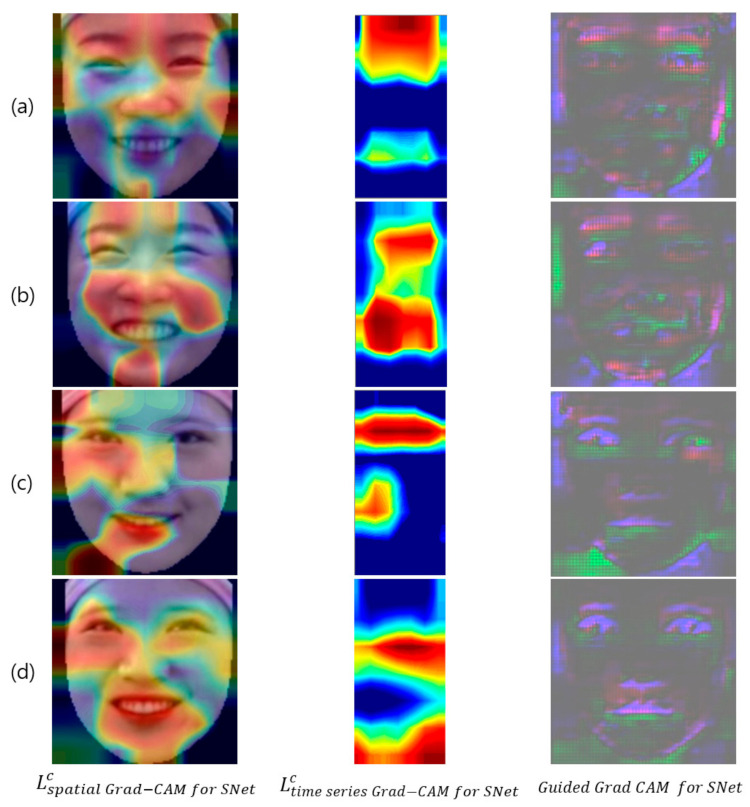
Visual demonstration of the proposed model. From left to right, $L_{spatial\;Grad-CAM\;for\;SNet}^{c}$, $L_{time\;series\;Grad-CAM\;for\;SNet}^{c}$, and Guided Grad CAM for SNet when the target class, c, is (**a**,**c**) a posed smile, and (**b**,**d**) a spontaneous smile.

$L_{Grad-CAM\;on\;R\;model}^{c}$ is the Grad-CAM for the right model, which is one of the pair of Siamese models, while the Grad-CAM result for the other pair is the left model, $L_{Grad-CAM\;on\;L\;model}^{c}$. Then, after obtaining the L1-norm distance between the two Grad-CAMs, $L_{Grad-CAM\;for\;SNet}^{c}$, the Grad-CAM for the Siamese architecture-based 3D CNN can be obtained through the absolute value function. We only used $L_{Grad-CAM\;for\;SNet}^{c}$ for the visual demonstration of the Siamese architecture-based 3D DenseNet, and applied it to $L_{spatial\;Grad-CAM}^{c}\;\mathrm{and}\;L_{time\;series\;Grad-CAM}^{c}$ in Equations (2) and (3), respectively. In addition, for visual assessment at high resolution, we checked the guided Grad-CAM. Guided Grad-CAM is a method of solving the low resolution problem of Grad-CAM ($7_{\left(\mathrm{image}\;\mathrm{width}\right)}\times 7_{\left(\mathrm{image}\;\mathrm{height}\right)})$ which combines the guided backpropagation ($112_{\left(\mathrm{image}\;\mathrm{width}\right)}\times 112_{\left(\mathrm{image}\;\mathrm{height}\right)})$ with Grad-CAM, with upsampling ($112_{\left(\mathrm{image}\;\mathrm{width}\right)}\times 112_{\left(\mathrm{image}\;\mathrm{height}\right)})$ (Selvaraju et al., 2017). Figure 4 presents the visual explanation of the proposed model, which confirms that the proposed model considers the spatial information around the eyes and lips, as well as the face boundaries, as the main features. In particular, movement around the eyes has been used as a major feature in the existing hand-crafted feature-based studies [12,19,20,21,22,23]. Furthermore, the upper region of the face is the main feature in both the time series and spatial domains, and the proposed model predicts spontaneous and posed smiles based on features of the spatiotemporal movements of the upper and lower region of the face.

### 4.3. Performance Comparison between 3D CNN Models with Diversiform Structures

**Figure 5:**
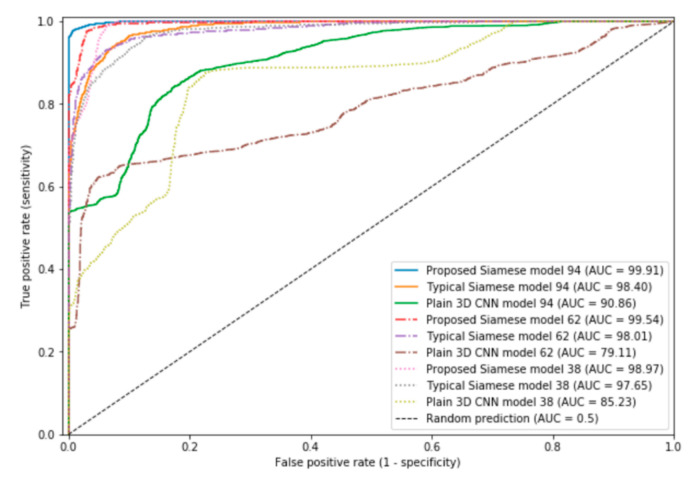
ROC curves of 3D CNN models with diversiform structures (AUC: area under curve).

We conducted experiments on three types of 3D CNN model structures for comparison: a posed smile anchor-based Siamese network (posed anchor Siamese Net), a neutral expression anchor-based Siamese network (neutral anchor Siamese Net), and the plain 3D CNN model. This section describes the main aspects of the performance comparison between the posed anchor Siamese Net, which received a genuine and imposter pair as input, and the neutral anchor Siamese Net which is the proposed model. Moreover, in the same way as the performance evaluation of the proposed model in Section 4.1, to conduct a more general evaluation, five-fold cross validation was performed for all 3D CNN model structures. Table 4 lists the performance measurement results for the three models with various network depths, and Figure 5 presents the receiver operating characteristic (ROC) curves of the 3D CNN models with diversiform structures. It is evident that the models with the Siamese network structure outperformed the plain 3D CNN model, and among the Siamese networks, the model using a neutral expression as an anchor exhibited the best performance. Thus, the neutral anchor Siamese Net has better classification performance than the posed anchor Siamese Net. However, the performance comparison in Table 4 alone cannot explain why the neutral anchor Siamese Net is superior to the posed anchor Siamese Net. To illustrate why the proposed model is better, in Section 4.4 we compare the visualizations from the deep learning model and the representational abilities of the feature vectors that constitute the output of the L1-norm distance layer.

### 4.4. Comparison between Neutral Anchor Siamese Net and Posed Anchor Siamese Net

**Figure 6:**
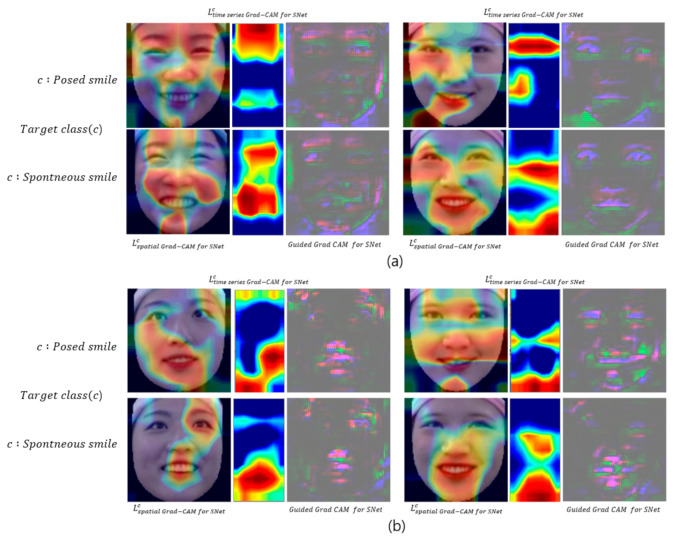
Visual feature comparison between neutral anchor Siamese Net and posed anchor Siamese Net, using $L_{spatial\;Grad-CAM\;for\;SNet}^{c}$, $L_{time\;series\;Grad-CAM\;for\;SNet}^{c}$, and Guided Grad CAM for SNet. (**a**) Visualization of neutral anchor Siamese Net (proposed method), when target class  c is a posed smile (first row), and a spontaneous smile (second row). (**b**) Visualization of posed anchor Siamese Net when target class, c, is a posed smile (first row), and a spontaneous smile (second row).

To compare these two models, we first analyzed the visual results of the deep learning model, using $L_{spatial\;Grad-CAM\;for\;SNet}^{c}$, $L_{time\;series\;Grad-CAM\;for\;SNet}^{c}$, and Guided Grad CAM for SNet, similarly to Section 4.2. Figure 6 shows the visual results obtained using these two models. In Figure 6a, the neutral anchor Siamese Net can be seen to have extracted spatiotemporal features from the entire face including the eyebrows. However, in Figure 6b, the activation maps are mainly distributed in the lower region of the face, and even in the guided Grad-CAM results, eyebrow features cannot be identified. Thus, the L1-norm distance of the spatiotemporal feature between the neutral expression and the two types of smile is greater than the L1-norm distance of the spatiotemporal feature between the posed smiles and the two types of smile, especially in the upper region of the face. This finding seems reasonable, because the spatiotemporal difference between the neutral expression and the two types of smile is greater than the spatiotemporal difference between the posed smile and the two types of smile. In particular, the movements of the upper region of the face, such as the eyebrow region, are small, and their spatiotemporal features are similar for different types of smile; therefore, the spatiotemporal difference may not be extracted from the L1-norm distance layer of the posed anchor Siamese Net. In other words, using a neutral expression as an anchor could be more suitable for extracting spatiotemporal metric features with the L1-norm distance of the Siamese network than using a posed smile as an anchor. Therefore, we compared and analyzed the representations of the L1-norm distance layer outputs of the two Siamese models.

**Figure 7:**
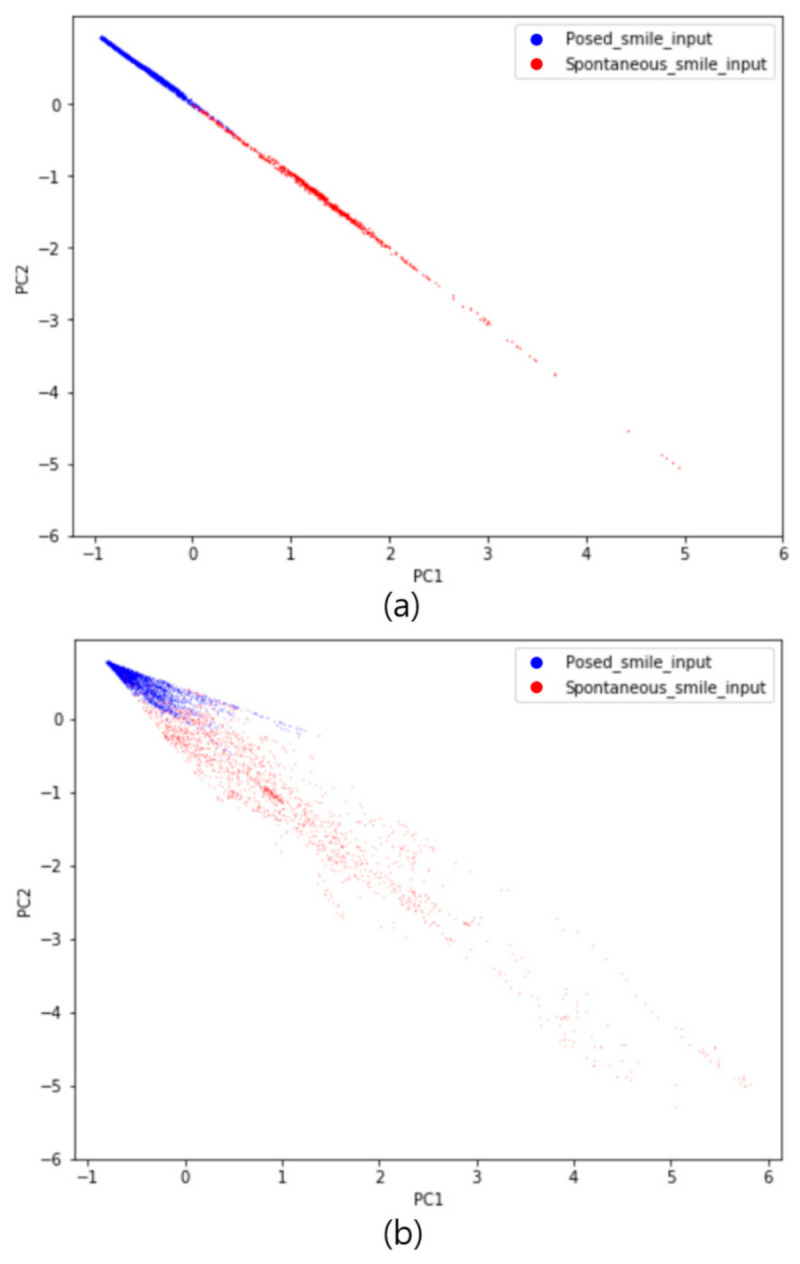
Euclidean representation of L1-norm distance, projected onto the top two singular vectors (PC1 and PC2). Euclidean representations of (**a**) neutral anchor Siamese Net (proposed method), and (**b**) posed anchor Siamese Net (conventional method).

Since the output of the L1-norm layer, a 1024-dimensional feature vector, is a spatiotemporal difference representation that can distinguish between spontaneous and posed smiles, we performed a comparative analysis focused on the L1-norm layer output. In addition, Figure 6 confirms that metric learning using a neutral expression as an anchor can provide a better spatiotemporal difference representation. We evaluated the discriminative power of the representation vectors by projecting a high-dimensional representation vector into a 2D Euclidean space through PCA, which was the method used by the authors who proposed the triplet network to compare its performance with that of a Siamese network [42]. Figure 7 compares the representation vectors of the two Siamese models. In Figure 7a, the posed and spontaneous smile inputs are both clustered in a specific 2D space. However, in Figure 7b, the posed smile inputs are clustered in a specific space, but the spontaneous smile inputs are widely spread. Visual analysis confirmed that the representation vectors of the neutral anchor Siamese Net could distinguish between the two types of smile more effectively than those of the posed anchor Siamese Net.

## 5. Discussion

In this study, we proposed a Siamese architecture-based 3D DenseNet using a neutral expression as an anchor for spontaneous and posed smile classification. A common Siamese network uses input pairs consisting only of posed and spontaneous smiles, but in the proposed method, input pairs consisting of a neutral expression and a posed smile, and a neutral expression and a spontaneous smile are used as input. We found that the classification performance of the Siamese network using a neutral expression as an anchor was higher than that of the conventional method. The difference between the two models is the composition of the input pair, as the common method is distinguished by learning through the similarity of genuine and imposter pairs. In the proposed method, the L1-norm distance between the neutral expression and two types of smile is used for deep metric learning. The performance measurement results demonstrated that the metric feature with a neutral expression as an anchor, which is used in the proposed method, is superior to the similarity feature of the common method.

To analyze why the proposed method performs smile classification better than the common method, we analyzed the visualization and L1-norm distance of the deep learning model. The visual analysis revealed that the distribution of the activation value of the proposed model appears around the eyes and lips, including the eyebrows, whereas in the case of the common method using a posed smile as the anchor, the activation value only appears in a portion of the face and not in the eyebrows. In particular, the visualizations obtained using the two models showed significant differences in the eyebrows and around the eyes, which is very important because the spatiotemporal features around the eyes have been used as the main features in related works [14,19,20,21,22,23,43]. This observation demonstrates that a more meaningful metric feature of the L1-norm distance layer can be extracted when using a neutral expression as an anchor than when using a genuine and imposter pair. Therefore, we employed PCA to compare and analyze the representations of the L1-norm distance values calculated using both methods. When the discriminative power of the L1-norm distance feature vector was confirmed by utilizing the top two singular vectors, the clustering of the feature vectors obtained using the proposed model was better. The results of this experiment confirmed that using a neutral expression as an anchor to extract the metric features of the two types of smile is more beneficial than performing similarity learning using a posed smile anchor as the input into the Siamese network. This is because a posed smile is similar to the two types of smile, and the ability to learn the similarity through the spatiotemporal features is limited. In contrast, it is considered that each type of smile can be mapped to the L1-norm distance feature space with a different subspace when using a neutral expression as an anchor. Due to the nature of the deep learning model, which is a black box, an analysis more detailed than that performed in these experiments would be difficult.

Most existing studies have attempted to classify spontaneous and posed smiles using hand-crafted approaches. Related studies using deep learning models have usually focused on methods using a single neural network, such as a CNN or DBM, and no previous researchers have attempted deep metric learning using a Siamese network for smile classification. In this study, we investigated a deep metric learning model for classifying two types of smile, using a Siamese network approach. We also suggested a neutral anchor Siamese Net for performance improvement. In addition, for comparison of the proposed method with the existing method, visual assessment, and representation analysis of the L1-norm distance vector using PCA were performed.

Although the proposed deep metric learning method exhibited a high performance, there were some limitations. The first limitation is related to the database built in this study. We wanted to use our database, as well as a public database, but public databases for deep learning methods are small and their availability is limited. Therefore, we only used our own database in this research. In related works, when analyzing the differences between spontaneous and posed smiles, or researching models for classifying two types of smile, the smile video was divided into three intervals, and each interval was analyzed separately. These three intervals are an onset period, during which the smile begins, an apex period, during which the smile is at its peak, and an offset period, during which the smile ends. Some studies have found that the onset is the most significant smile interval for spontaneous and posed classification [12]. Following the methods of previous studies, we had to divide the smile into onset, apex, and offset intervals to train the classification model, but the number of data were insufficient for training our proposed model. Therefore, we only performed time-series segmentation for entire smile intervals. Although some existing hand-crafted feature-based classification models have shown higher classification accuracy when using entire smile intervals, we believe that it is also meaningful to confirm significant difference between each smile interval in deep metric learning, as future work can collect more data. The second limitation is that the nature of deep metric learning models as black boxes restricts their analysis. Although visualization using Grad-CAM, and an analysis of the discriminative power of the L1-norm distance vector using PCA, were utilized in this study to explain the superiority of the proposed method to the existing method, a detailed analysis was not possible. The reason for the high discriminative power of the L1-norm distance vector of the proposed method, could be that the distance between a neutral expression and the two types of smile is larger than the distance between a posed smile and the two types of smile; however, this is only an inference. These limitations should be addressed in future works. We also confirmed that Grad-CAM is correlated with the key features identified in previous studies. This finding suggests that class activation maps of deep learning models can be applied for feature extraction and explanation in future works involving facial expressions. In addition, due to the recent rapid development of imaging spectrometers, and the advance of image processing and deep learning models, we believe that further research is also needed into high-dimensional image classification technology for facial expression analysis by performing more abundant feature extraction through spectral unmixing [44].

## 6. Conclusions

In this study, we proposed a Siamese architecture-based 3D DenseNet model to serve as a deep metric learning method for spontaneous and posed smile classification. It was confirmed that the L1-norm distance features of the two types of smile provided better performance when learning was conducted using a neutral expression as an anchor than when input consisting of genuine and imposter pairs was used for learning, as in the conventional method. The classification accuracy of the best proposed model was 98.12%. To analyze the performance of the proposed method, which was higher than that of the existing method, we designed Grad-CAM that can be applied to a Siamese architecture-based 3D CNN, and compared the discriminative power of the L1-norm distance feature vectors using PCA. When a neutral expression was used as an anchor, the model extracted higher discriminative features from the entire face, including the eyebrows. In conclusion, the proposed method using a neural expression as an anchor not only provided high performance through person-specific normalization, but also achieved higher performance than the existing Siamese model method using genuine and imposter pairs. Furthermore, the proposed method will facilitate full automation in practical applications, since expert intervention is required to use a posed or spontaneous smile as an anchor, but not when using a neutral expression as an anchor.

## Figures and Tables

**Table 1:** Details of datasets after data preprocessing.

	Number of Subjects	Number of Spontaneous Smile Data (%)	Number of Posed Smile Data (%)	Number of Total Data (%)
Training set	68	18,300 (75%)	23,486 (77%)	41,786 (76%)
Validation set	10	1719 (7%)	3753 (12%)	5472 (10%)
Test set	10	4473 (18%)	3261 (11%)	7734 (14%)
Total	88	24,492 (100%)	30,500 (100%)	54,992 (100%)

**Table 2:** Model specification of the 3D DenseNet based on Siamese architecture.

Layers	Output Size (Width × Height × Time Series)	3D DenseNet Based on Siamese Architecture-60
3D convolution	56 × 56 × 30	3×3×3 convolution, 2×2×1 stride
3D dense block (1)	56 × 56 × 30	$\left[\;\begin{array}{c} 1\times 1\times 1\;\mathrm{convolution}\\ 3\times 3\times 3\;\mathrm{convolution} \end{array}\;\right]\times 3$
Transition layer (1)	56 × 56 × 30	1×1×1 convolution,1×1×1 stride
	28 × 28 × 15	2×2×2 average pooling, 2×2×2 stride
3D dense block (2)	28 × 28 × 15	$\left[\;\begin{array}{c} 1\times 1\times 1\;\mathrm{convolution}\\ 3\times 3\times 3\;\mathrm{convolution} \end{array}\;\right]\times 12$
Transition layer (2)	28 × 28 × 15	1×1×1 convolution,1×1×1 stride
	14 × 14 × 15	2×2×2 average pooling, 2×2×1 stride
3D dense block (3)	14 × 14 × 15	$\left[\;\begin{array}{c} 1\times 1\times 1\;\mathrm{convolution}\\ 3\times 3\times 3\;\mathrm{convolution} \end{array}\;\right]\times 12$
Transition layer (3)	14 × 14 × 15	1×1×1 convolution,1×1×1 stride
	7 × 7 × 7	2×2×2 average pooling, 2×2×2 stride
3D dense block (4)	7 × 7 × 7	$\left[\;\begin{array}{c} 1\times 1\times 1\;\mathrm{convolution}\\ 3\times 3\times 3\;\mathrm{convolution} \end{array}\;\right]\times 12$
3D convolution	7 × 7 × 7	1×1×1 convolution,1×1×1 stride

In transition layer (2), pooling reduces the width and height of the output without reducing its depth.

**Table 3:** Performance of the proposed model.

Network	Accuracy	Precision	Recall	AUC
Lee et al. [39]	87.13%	90.15%	86.94%	87.99%
3D DenseNet-38	97.06%	97.21%	98.53%	98.97%
3D DenseNet-62	95.87%	98.84%	95.10%	99.54%
3D DenseNet-94	98.12%	97.85%	99.44%	99.91%

AUC: area under curve.

**Table 4:** Performance of 3D CNN models with diversiform structures.

Model	Network Depth	Accuracy	Precision	Recall	AUC
Neutral anchor Siamese Net (proposed model)	38	97.06%	97.21%	98.53%	98.97%
	62	95.87%	98.84%	95.10%	99.54%
	94	98.12%	97.85%	99.44%	99.91%
Posed anchor Siamese Net	38	91.31%	95.85%	91.16%	97.65%
	62	93.73%	95.20%	95.59%	98.01%
	94	93.08%	92.01%	98.36%	98.40%
Plain 3D CNN	38	65.86%	89.39%	56.99%	85.23%
	62	72.77%	92.52%	65.60%	79.11%
	94	71.24%	93.91%	62.08%	90.86%

AUC: area under curve.

## References

[1] Bibri S.E. (2015). The Human Face of Ambient Intelligence.

[2] Duthoit C.J., Sztynda T., Lal S.K., Jap B.T., Agbinya J.I. (2008). Optical flow image analysis of facial expressions of human emotion: Forensic applications. Proceedings of the 1st International Conference on Forensic Applications and Techniques in Telecommunications, Information, and Multimedia and Workshop.

[3] Haamer R.E., Kulkarni K., Imanpour N., Haque M.A., Avots E., Breisch M., Naghsh-Nilchi A.R. (2018). Changes in facial expression as biometric: A database and benchmarks of identification. Proceedings of the 13th IEEE International Conference on Automatic Face & Gesture Recognition.

[4] Manfredonia J., Bangerter A., Manyakov N.V., Ness S., Lewin D., Skalkin A., Leventhal B. (2019). Automatic recognition of posed facial expression of emotion in individuals with autism spectrum disorder. J. Autism Dev. Disord..

[5] Adams B., Garrido C.O., Albohn D.N., Hess U., Kleck R.E. (2016). What facial appearance reveals over time: When perceived expressions in neutral faces reveal stable emotion dispositions. Front. Psychol..

[6] Li S., Deng W. (2020). Deep facial expression recognition: A survey. IEEE Trans. Affect. Comput..

[7] Caltagirone C., Ekman P., Friesen W., Gainotti G., Mammucari A., Pizzamiglio L., Zoccolotti P. (1989). Posed emotional expression in unilateral brain damaged patients. Cortex.

[8] Adolphs R. (2002). Recognizing emotion from facial expressions: Psychological and neurological mechanisms. Behav. Cogn. Neurosci. Rev..

[9] Jankovic J. (2008). Parkinson’s disease: Clinical features and diagnosis. J. Neurol. Neurosurg. Psychiatry.

[10] Smith M.C., Smith M.K., Ellgring H. (1996). Spontaneous and posed facial expression in Parkinson’s disease. J. Int. Neuropsychol. Soc..

[11] Ekman P., Hager J.C., Friesen W.V. (1981). The symmetry of emotional and deliberate facial actions. Psychophysiology.

[12] Ross E.D., Pulusu V.K. (2013). Posed versus spontaneous facial expressions are modulated by opposite cerebral hemispheres. Cortex.

[13] Borod J.C., Koff E., Lorch M.P., Nicholas M. (1986). The expression and perception of facial emotion in brain-damaged patients. Neuropsychologia.

[14] Gazzaniga M.S., Smylie C.S. (1990). Hemispheric mechanisms controlling voluntary and spontaneous facial expressions. J. Cogn. Neurosci..

[15] Ji S., Xu W., Yang M., Yu K. (2012). 3D convolutional neural networks for human action recognition. IEEE Trans. Pattern Anal. Mach. Intell..

[16] Koch G., Zemel R., Salakhutdinov R. (2015). Siamese Neural Networks for One-Shot Image Recognition. Master’s Thesis.

[17] Rasti B., Hong D., Hang R., Ghamisi P., Kang X., Chanussot J., Benediktsson J.A. (2020). Feature extraction for hyperspectral imagery: The evolution from shallow to deep. arXiv.

[18] Oh S.H., Xiang Y., Jegelka S., Savarese S. Deep metric learning via lifted structured feature embedding. Proceedings of the IEEE Conference on Computer Vision and Pattern Recognition.

[19] Dibeklioglu H., Valenti R., Salah A.A., Gevers T. Eyes do not lie: Spontaneous versus posed smiles. Proceedings of the 18th ACM international conference on Multimedia.

[20] Duchenne G.B., de Boulogne G.B.D. (1990). Duchenne and facial expression of emotion. The Mechanism of Human Facial Expression.

[21] Dibeklioğlu H., Salah A.A., Gevers T. (2015). Recognition of genuine smiles. IEEE Trans. Multimed..

[22] Valstar M.F., Gunes H., Pantic M. (2007). How to distinguish posed from spontaneous smiles using geometric features. Proceedings of the 9th International Conference on Multimodal Interfaces.

[23] Wu P.P., Liu H., Zhang X.W., Gao Y. (2017). Spontaneous versus posed smile recognition via region-specific texture descriptor and geometric facial dynamics. Front. Inf. Technol. Electron. Eng..

[24] FERC 2013 Form 714—Annual Electric Balancing Authority Area and Planning Area Report (Part 3 Schedule 2). Form 714 Database, Federal Energy Regulatory Commission, 2013; pp. 2006–2012. https://datarepository.wolframcloud.com/resources/FER-2013.

[25] Mandal B., Lee D., Ouarti N. (2016). Distinguishing Posed and Spontaneous Smiles by Facial Dynamics. Computer Vision—ACCV 2016.

[26] Parkhi O.M., Vedaldi A., Zisserman A. Deep face recognition. Proceedings of the British Machine Vision Conference (BMVC).

[27] Ojansivu V., Heikkil J. (2008). Blur insensitive texture classification using local phase quantization. Image Signal. Process..

[28] Dalal N., Triggs B. Histograms of oriented gradients for human detection. Proceedings of the 2005 IEEE Computer Society Conference on Computer Vision and Pattern Recognition (CVPR’05).

[29] Farneback G. Two-frame motion estimation based on polynomial expansion. Proceedings of the SCIA’03, the 13th Scandinavian Conference on Image Analysis.

[30] Gan Q., Wu C., Wang S., Ji Q. Posed and spontaneous facial expression differentiation using deep Boltzmann machines. Proceedings of the 2015 International Conference on Affective Computing and Intelligent Interaction (ACII).

[31] Wang S., Liu Z., Lv S., Lv Y., Wu G., Peng P., Wang X. (2010). A natural visible and infrared facial expression database for expression recognition and emotion inference. IEEE Trans. Multimed..

[32] Pfister T., Li X., Zhao G., Pietikainen M. (2011). Differentiating spontaneous from posed facial expressions within a generic facial expression recognition framework. Proceedings of the Computer Vision Workshops (ICCV Workshops).

[33] Kumar G.R., Kumar R.K., Sanyal G. Discriminating real from fake smile using convolution neural network. Proceedings of the 2017 International Conference on Computational Intelligence in Data Science (ICCIDS) IEEE.

[34] Valstar M., Pantic M. Induced disgust, happiness and surprise: An addition to the MMI facial expression database. Proceedings of the 3rd International Workshop on EMOTION (satellite of LREC): Corpora for Research on Emotion and Affect.

[35] Yang Y., Hossain M.Z., Gedeon T., Rahman S. RealSmileNet: A Deep End-To-End Network for Spontaneous and Posed Smile Recognition. Proceedings of the 15th Asian Conference on Computer Vision.

[36] Shi X., Chen Z., Wang H., Yeung D.Y., Wong W.K., Woo W.C. (2015). Convolutional LSTM network: A machine learning approach for precipitation nowcasting. Adv. Neural Inf. Process. Syst..

[37] Wang S., Hao L., Ji Q. (2020). Posed and Spontaneous Expression Distinction Using Latent Regression Bayesian Networks. ACM Trans. Multimed. Comput. Commun. Appl..

[38] Baltrušaitis T., Mahmoud M., Robinson P. Cross-dataset learning and person-specific normalisation for automatic action unit detection. Proceedings of the 11th IEEE International Conference and Workshops on Automatic Face and Gesture Recognition (FG).

[39] Lee K., Lee E.C. (2018). Facial asymmetry feature based spontaneous facial expression classification using temporal convolutional networks and support vector regression. Basic Clin. Pharmacol. Toxicol..

[40] Simonyan K., Zisserman A. (2014). Two-stream convolutional networks for action recognition in videos. Adv. Neural Inf. Process. Syst..

[41] Selvaraju R.R., Cogswell M., Das A., Vedantam R., Parikh D., Batra D. Grad-cam: Visual explanations from deep networks via gradient-based localization. Proceedings of the IEEE International Conference on Computer Vision.

[42] Hoffer E., Ailon N., Feragen A., Pelillo M., Loog M. (2015). Deep metric learning using triplet network. Similarity-Based Pattern Recognition. SIMBAD 2015. Lecture Notes in Computer Science.

[43] Park S., Lee K., Lim J.A., Ko H., Kim T., Lee J.I., Lee J.Y. (2020). Differences in Facial Expressions between Spontaneous and Posed Smiles: Automated Method by Action Units and Three-Dimensional Facial Landmarks. Sensors.

[44] Hong D., Yokoya N., Chanussot J., Zhu X.X. (2018). An augmented linear mixing model to address spectral variability for hyperspectral unmixing. IEEE Trans. Image Process..

